# Identification of long non-coding RNAs GAS5, linc0597 and lnc-DC in plasma as novel biomarkers for systemic lupus erythematosus

**DOI:** 10.18632/oncotarget.15569

**Published:** 2017-02-21

**Authors:** Guo-Cui Wu, Jun Li, Rui-Xue Leng, Xiang-Pei Li, Xiao-Mei Li, De-Guang Wang, Hai-Feng Pan, Dong-Qing Ye

**Affiliations:** ^1^ Department of Epidemiology and Biostatistics, School of Public Health, Anhui Medical University, Hefei, Anhui, China; ^2^ The Key Laboratory of Major Autoimmune Diseases, Anhui, China; ^3^ Department of Rheumatology and Immunology, Anhui Provincial Hospital, Hefei, Anhui, China; ^4^ Department of Nephrology, The Second Affiliated Hospital of Anhui Medical University, Hefei, Anhui, China

**Keywords:** biomarker, diagnosis, long non-coding RNA, systemic lupus erythematosus

## Abstract

Despite increasing evidence that long non-coding RNAs (lncRNAs) widely take part in human diseases, the role of lncRNAs in systemic lupus erythematosus (SLE) is largely unknown. In this study, we performed a two-stage study to explore the plasma levels of five lncRNAs (GAS5, linc0949, linc0597, HOTAIRM1 and lnc-DC) and their potential as SLE biomarkers. Compared with healthy controls, plasma levels of GAS5 and lnc-DC were significantly decreased (*P* < 0.001 and *P* = 0.002, respectively) while linc0597 were overexpressed in SLE patients (*P* < 0.001). When SLE patients were divided into SLE without nephritis and lupus nephritis (LN), the levels of lnc-DC were significantly higher in LN compared with SLE without nephritis (*P* = 0.018), but no significant difference in levels of GAS5 and linc0597 were found between LN and SLE without nephritis; plasma linc0949 level showed no significant difference in all comparisons. Further evaluation on potential biomarkers showed that GAS5, linc0597 and lnc-DC may specifically identify patients with SLE, the combination of GAS5 and linc0597 provided better diagnostic accuracy; lnc-DC may discriminate LN from SLE without nephritis. In summary, GAS5, linc0597 and lnc-DC in plasma could be potential biomarkers for SLE.

## INTRODUCTION

Systemic lupus erythematosus (SLE) is a complicated autoimmune disease characterized by the production of multiple autoantibodies, immune-complex deposition and excessive proinflammatory cytokine, resulting in multiple organ systems damages [1, 2]. One of the most severe manifestations of SLE is lupus nephritis (LN), which is a major cause of substantial morbidity and mortality [3, 4]. Due to the heterogeneous presentation of SLE patients and their unpredictable disease course, it is essential to explore novel biomarkers that will contribute to better diagnosis and prognosis.

Despite the great efforts on the research of molecular alterations involved in SLE [5–8], the pathogenesis of SLE is still incompletely elucidated. Thus, in-depth research on the genetic and molecular aberrations of SLE is still an urgent issue and likely to be the key for identifying novel biomarkers. More than 80% of the human genome is transcribed into RNA transcripts without protein-coding potential [9]. According to the transcript size, these so-called noncoding RNAs (ncRNAs) are loosely divided into two major classes: small ncRNAs (<200 nt), such as microRNAs (miRNAs), and long ncRNAs (≥200 nt). Several studies have revealed the potential role of miRNAs in the pathogenesis of SLE and as biomarkers for the diagnosis of SLE [10–13]. Emerging evidences have demonstrated that long non-coding RNAs (lncRNAs) can regulate gene expression and widely participate in normal physiological and disease conditions. However, the role of lncRNAs in SLE is largely unknown.

Recently, a number of lncRNAs have been reported to be involved in the pathogenesis of immune-mediated inflammatory diseases like SLE [14–17]. A long noncoding RNA gene, growth arrest specific 5 (GAS5), has been linked with increased susceptibility of SLE in mouse model [18]. Moreover, GAS5 as a prime candidate for the chromosome 1q25 SLE locus, has been shown to be related with human SLE development in genetic studies [19, 20]. Recently, Wu *et al* reported two lncRNAs, linc0949 and linc0597, were significantly decreased in the peripheral blood mononuclear cells (PBMCs) of SLE patients, and linc0949 could serve as a biomarker for diagnosis, disease activity evaluation and therapeutic response in SLE [15]. Furthermore, recent studies have revealed that lncRNAs are involved in immune cell development, such as granulocytes, dendritic cells (DCs), *etc*. [14]. HOX antisense intergenic RNA myeloid 1 (HOTAIRM1), as a myeloid-specific lincRNA, is located at 3′ end of the HOXA cluster and plays a key role in granulocyte maturation [21, 22]. A very recent study showed that PU.1, which has been implicated in SLE pathogenesis, controls the expression of HOTAIRM1 during granulocytic differentiation [23]. Wang *et al* identified an intergenic lncRNAs, named lnc-DC, is exclusively expressed in DCs, and supports capacity of DCs to stimulate T cell activation [24], thereby may play a role in the pathogenesis of SLE.

Circulating RNA in plasma or serum has been an emerging field for noninvasive diagnostic application. Available evidence have demonstrated that lncRNAs are stable in human plasma. Several studies in cancer and cardiovascular diseases have demonstrated that circulating cell-free lncRNAs could be acted as biomarkers for these diseases [25–30]. However, circulating cell-free lncRNAs expression signatures in SLE patients has never been examined. We hypothesized that there are specific circulating cell-free lncRNAs that may serve as SLE biomarkers. In the present study, we performed a two-stage study to explore the plasma levels of five lncRNAs (GAS5, linc0949, linc0597, HOTAIRM1 and lnc-DC) and their potential as biomarkers in SLE.

## RESULTS

### Characteristics of study subjects in the first stage screening and second stage validation

The demographic and baseline clinical data of the study subjects in the two stages are summarized in Table 1.

**Table 1:** Characteristics of study subjects in the screening and validation stage

Characteristics	Screening stage	Validation stage
**Control subjects**	12	80
Age (year)	25±2	29 (26, 36)
Sex (female/male)	12/0	75/5
**Patients with SLE**	24	163
Age (year)	28±6	34 (25, 47)
Sex (female/male)	23/1	150/13
Disease duration (year)	0	4 (1, 8)
SLEDAI-2K	14±8	13 (8, 20)
Disease manifestations		
Renal disease	12 (50)	65 (40)
Vasculitis	2 (8)	6 (4)
Arthritis	8 (33)	61 (37)
Myositis	2 (8)	14 (9)
Rash	15 (63)	79 (48)
Alopecia	10 (42)	68 (42)
Oral ulcer	5 (21)	29 (18)
Pleuritis	0	16 (10)
Leukopenia	6 (25)	26 (16)
Thrombocytopenia	5 (21)	46 (28)
Fever	10 (42)	47 (29)
Nervous system disorder	4 (17)	43 (26)
Low complement	21 (88)	110 (67)*
Autoantibodies		
Anti-dsDNA	16 (67)	71(44)*
Anti-Sm	14 (58)	54 (34)*
Anti-SSA	20 (83)	111 (70)
Anti-SSB	7 (29)	24 (15)
Anti-RNP	18 (75)	62(39)^*^
Anti-Ribosomal P	12 (50)	47 (30)
Medical therapy		
Prednisone dose ≥15 mg/day	0	81 (50)^**^*
Prednisone dose <15 mg/day	0	71 (44)^**^*
Antimalarials	0	126 (77)^**^*
Azathioprine, MTX, or CTX	0	33 (20)*

Normally distributed data were expressed as means ± standard deviation (SD), variables with a skewed distribution were presented as median (interquartile range).

Categorical variables values were described as number (%).

SLEDAI-2K: Systemic Lupus Erythematosus Disease Activity Index 2000; dsDNA: double-stranded DNA; Sm: Smith; SSA: Sjögren's syndrome-related antigen A; SSB: Sjögren's syndrome-related antigen B; RNP: Ribonucleoprotein; MTX: methotrexate; CTX: cyclophosphamide

*P<0.05; ** *P*<0.01; ***P<0.001.

### Screening of five selected lncRNAs in plasma of SLE and healthy subjects

The five selected lncRNAs (GAS5, linc0949, linc0597, HOTAIRM1, and lnc-DC) were first tested in plasma from 24 new-onset patients with SLE and 12 healthy controls using qRT-PCR. Compared with healthy controls, the expression level of GAS5 were significantly down-regulated (*P* <0.001) while linc0597 were significantly overexpressed with >2-fold change in all patients with SLE (*P* = 0.011). No significant differences in other three lncRNAs were found between SLE patients and healthy controls (all *P* >0.05).

When SLE patients were divided into LN and SLE without nephritis, the results showed that the levels of GAS5 were also significantly down-regulated in both SLE patients with and without nephritis relative to healthy controls (*P* = 0.022 and *P* < 0.001, respectively); compared with healthy controls, the level of linc0949 was overexpressed in SLE without nephritis, while significantly down-regulated in LN (both *P* < 0.001); the levels of lnc-DC were significantly higher in LN, while significantly decreased in SLE without nephritis (both *P* < 0.001); the level of linc0597 was only overexpressed in SLE without nephritis (*P* < 0.001); however, the plasma levels of HOTAIRM1 showed no significant difference between groups and was therefore excluded from the second stage validation (Table 2).

**Table 2:** Comparison of plasma lncRNAs levels between different subgroups in the screening stage

Group	Number	GAS5	Linc0949	Linc0597	HOTAIRM1	Lnc-DC
Healthy controls	12	0.96(0.87, 1.08)	0.98(0.85, 1.19)	0.99(0.80, 1.18)	0.79(0.63, 1.34)	0.88(0.73, 1.33)
SLE patients	24	0.40(0.19,0.79) ^**^*	0.97(0.26, 2.12)	1.90(1.17, 2.46)*	0.97(0.73, 1.30)	1.05(0.27, 2.34)
SLE without nephritis	12	0.40(0.23, 0.62)^**^*	2.07(1.67, 2.32)^**^*	2.01(1.44, 2.22)^**^*	1.01(0.73, 1.35)	0.27(0.23, 0.30)^**^*
SLE with nephritis	12	0.48(0.18, 1.10)*	0.27(0.17, 0.38)^**^*	1.41(0.50, 4.97)	0.97(0.65, 1.28)	2.34(2.05, 3.00)^**^*

Median (interquartile range)

^**^* *vs* healthy controls *P*< 0.001

^*^ *vs* healthy controls *P*< 0.01

* *vs* healthy controls *P*< 0.05

### Validation of candidate lncRNAs identified in the first stage screening in plasma of SLE and healthy subjects

**Figure 1:**
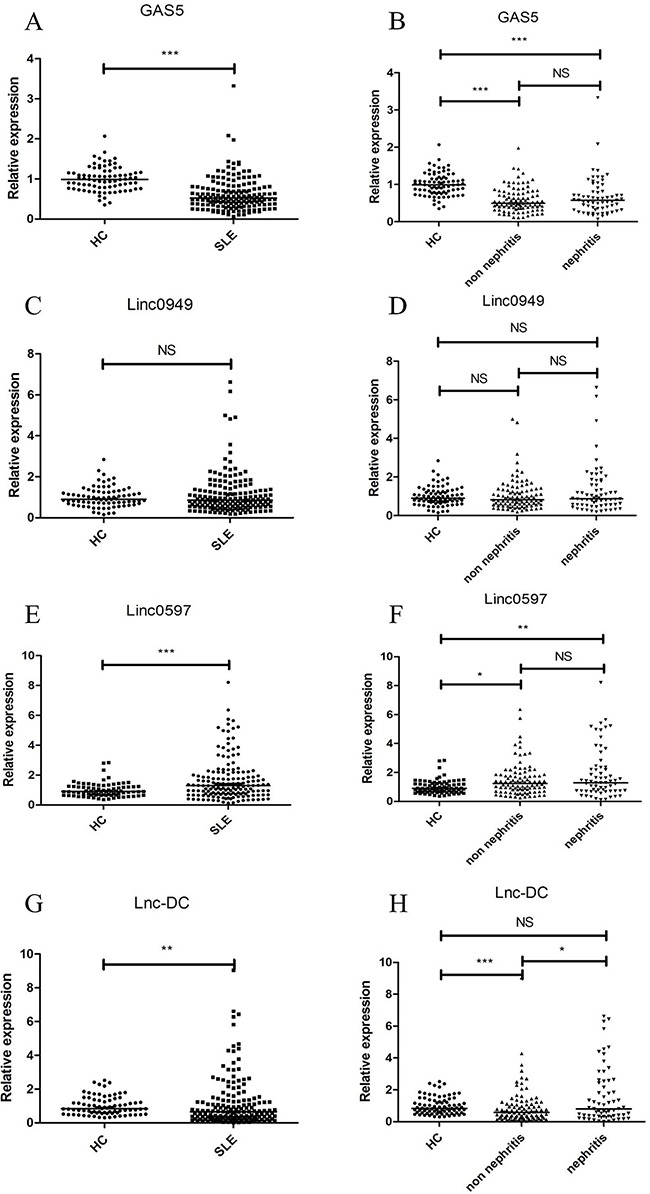
Validation of candidate lncRNAs (GAS5, linc0949, linc0597 and lnc-DC) identified in the first stage screening in plasma of SLE and healthy subjects Each symbol represents an individual subjects; horizontal lines indicate median values. The expression levels of the four candidate lncRNAs in 163 SLE patients, 80 healthy controls were analyzed by qRT-PCR and normalized by GAPDH. **(A)** Decreased expression of GAS5 in total SLE patients *vs* healthy controls. **(B)** Decreased expression of GAS5 in both SLE patients with and without nephritis *vs* healthy controls, but no significant difference in GAS5 between SLE patients with and without nephritis. **(C)** No significant difference in linc0949 between total SLE patients and healthy controls. **(D)** No significant difference in linc0949 among healthy controls, SLE patients with and without nephritis. **(E)** Increased expression of linc0597 in total SLE patients *vs* healthy controls. **(F)** Increased expression of linc0597 in both SLE patients with and without nephritis *vs* healthy controls, but no significant difference in linc0597 between SLE patients with and without nephritis. **(G)** Decreased expression of lnc-DC in total SLE patients *vs* healthy controls. **(H)** Decreased expression of lnc-DC in SLE patients without nephritis *vs* healthy controls and in SLE patients without nephritis *vs* SLE patients with nephritis, but no significant difference in lnc-DC in SLE patients with nephritis *vs* healthy controls. SLE: systemic lupus erythematosus; HC: healthy controls; NS: not significant; ^**^* *P*< 0.001, ^*^ *P*< 0.01, * *P*< 0.05.

To verify the results of four candidate lncRNAs (GAS5, linc0949, linc0597 and lnc-DC) identified in the screening stage and to assess their potential value as a signature of SLE or LN, we conducted a validation study in an independent cohort including 163 SLE patients and 80 healthy controls. As shown in Figure 1, the expression levels of GAS5, linc0597 and linc0949 were consistent with the results from the screening stage. GAS5 and linc0597 were also significantly altered in all SLE patients compared with the healthy controls (both *P* < 0.001), and level of linc0949 also showed no significant difference. However, different from the screening stage, the level of lnc-DC was significantly down-regulated in all SLE patients as compared with healthy controls (*P* = 0.002).

When SLE patients were divided into LN and SLE without nephritis, compared with healthy controls, the levels of GAS5 were significantly lower in both SLE patients with and without nephritis (both *P* < 0.001), the levels of linc0597 were significantly higher in both SLE patients with and without nephritis (*P* =0.003 and *P* =0.017, respectively), no significant difference in levels of GAS5 and linc0597 were found between patients with LN and SLE without nephritis; the levels of lnc-DC were down-regulated in SLE without nephritis compared with healthy controls (*P* < 0.001), and significantly up-regulated in patients with LN when compared with SLE without nephritis (*P* = 0.018); however, in this assay the levels of linc0949 showed no significant difference in all comparisons.

### Association of plasma GAS5, linc0597, lnc-DC and linc0949 levels with SLE clinical characteristics

Supplementary Table 2–3 shows the correlation between plasma levels of GAS5, linc0597, lnc-DC, linc0949 and the clinical characteristics of all patients with SLE. GAS5 level was significantly lower in more active SLE patients than in less active SLE patients (*P* = 0.003). In addition, GAS5 level was negatively associated with SLEDAI-2K score in patients with SLE (*r* = −0.143, *P* = 0.051). Moreover, plasma level of GAS5 was also negatively correlated with Erythrocyte Sedimentation Rate (ESR) (*r* = −0.230, *P* = 0.002).

Linc0597 expression was significantly increased in SLE patients with hypocomplementemia compared with those with normal levels of complements (*P* = 0.029). Further analysis revealed a negative correlation between linc0597 level and complements 3 (C3) level (*r* = -0.263, *P* < 0.001). Moreover, lnc-DC also had weakly negative correlation with C3 level (*r* = -0.173, *P* = 0.020).

Although levels of linc0949 showed no significant difference between SLE patients and healthy controls, as well as LN and SLE without nephritis, association analysis showed that linc0949 was significantly lower in more active SLE patients than in less active SLE patients (*P* = 0.020). In addition, a negative correlation between linc0949 and SLEDAI-2K score was observed (*r* = -0.166, *P* = 0.023). Moreover, level of linc0949 was also significantly lower in SLE patients with positive anti-double stranded DNA (dsDNA) than those without (*P* = 0.023), and it also had weakly negative correlation with C3 level (*r* = -0.158, *P* = 0.034).

When medical therapies were considered, expression of GAS5, linc0597, lnc-DC and linc0949 showed no significant difference between SLE patients who have received prednisone treatment or not. Then the SLE patients treated with prednisone were divided into patients receiving medium to high doses of prednisone (>15 mg/day) and low doses of prednisone (≤15 mg/day), no significant differences were observed either. Besides, expression of GAS5, linc0597 and lnc-DC and linc0949 was compared between SLE patients who have not receiving immunosuppressive treatment and those receiving treatment with any of the following: azathioprine, methotrexate (MTX); cyclophosphamide (CTX) or hydroxychloroquine, but no significant differences were observed.

### Identification of lncRNAs in plasma as novel biomarkers for SLE

**Figure 2:**
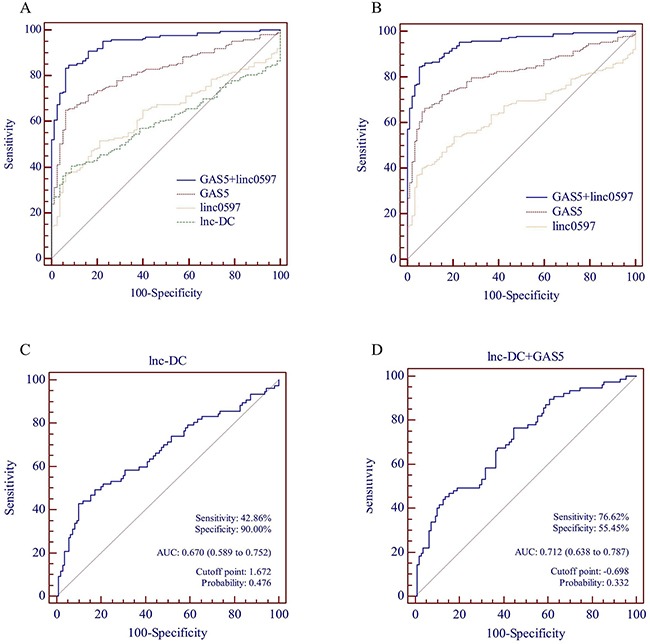
Receiver operating characteristic (ROC) curve analysis of lncRNAs for the discriminative ability of SLE patients *vs* healthy controls and SLE with nephritis *vs* SLE without nephritis **(A)** lnc-DC alone, linc0597 alone, GAS5 alone and GAS5 combined with linc0597 for the discriminative ability of SLE patients *vs* healthy controls in the validation set. **(B)** linc0597 alone, GAS5 alone and GAS5 combined with linc0597 for the discriminative ability of SLE patients *vs* healthy controls in the combination set. **(C)** lnc-DC alone for the discriminative ability of SLE with nephritis *vs* SLE without nephritis in the combination set. **(D)** lnc-DC combined with GAS5 for the discriminative ability of SLE with nephritis *vs* SLE without nephritis in the combination set.

To further evaluate the potential of the three lncRNAs (GAS5, linc0597 and lnc-DC) identified in the validation stage in plasma as novel biomarkers for SLE, ROC curves analysis were performed on data from the validation set and the combination of screening set and validation set (Figure 2 and Table 3).

**Table 3:** Performance of GAS5, linc0597 and lnc-DC in the differential diagnosis SLE patients from healthy controls

Stage	lncRNA	AUC	Sensitivity	Specificity	Accuracy	PPV	NPV	Cutoff point	Probability
Validation set (SLE = 163 and HC = 80)	GAS5	0.819	65.03%	93.75%	74.49%	0.37%	99.99%	0.648	0.748
	linc0597	0.637	36.81%	93.75%	55.56%	0.21%	99.98%	1.584	0.726
	lnc-DC	0.600	40.49%	91.25%	57.20%	0.17%	99.98%	0.423	0.654
	GAS5+ linc0597	0.942	83.44%	93.75%	86.83%	0.48%	99.99%	0.981	0.727
Combination set (SLE = 187 and HC = 92)	GAS5	0.819	66.31%	92.39%	74.91%	0.31%	99.99%	0.657	0.739
	linc0597	0.652	40.11%	93.48%	57.71%	0.22%	99.98%	1.584	0.728
	GAS5+ linc0597	0.948	84.49%	94.57%	87.81%	0.56%	99.99%	0.942	0.719

AUC: the area under curve; PPV: positive predictive value; NPV: negative predictive value

From the data of validation set, the AUC of the ROC curve was 0.819 (95% CI: 0.766-0.871; *P* < 0.001) for GAS5, 0.637 (95% CI: 0.569-0.705; *P* = 0.001) for linc0597, 0.600 (95% CI: 0.532-0.669; *P* = 0.011) for lnc-DC, respectively. Further analysis of the diagnostic performance of GAS5, linc0597 and lnc-DC revealed that, plasma level of GAS5 could distinguish SLE from healthy controls with 65.03% sensitivity and 93.75% specificity, though the plasma levels of linc0597 and lnc-DC were less sensitive (36.81% and 40.49%) for SLE detection. These results indicated that GAS5 may be a promising biomarker for SLE diagnosis. To evaluate the cumulative performances of the three lncRNAs in discriminating SLE from healthy controls, a binary logistic regression was performed. The logistic regression model showed that combination of GAS5 and linc0597 could provide better diagnostic accuracy, with the AUC of 0.942 (95% CI: 0.913-0.970; *P* < 0.001), which was considerably higher than the AUC of GAS5 (0.819; *P* < 0.001). The sensitivity and specificity of the combination of GAS5 and linc0597 were 83.44% and 93.75%, respectively.

Then we further evaluated the combination of GAS5 and linc0597 as biomarkers for SLE in 187 patients with SLE and 92 healthy controls. The AUC of GAS5 was 0.819 (95% CI: 0.770-0.868; *P* < 0.001; sensitivity = 66.31%, specificity = 92.39%) and linc0597 was 0.652 (95% CI: 0.589-0.715; *P* < 0.001; sensitivity = 40.11%, specificity = 93.48%), and it was 0.948 (95% CI: 0.924-0.972; *P* < 0.001) when GAS5 and linc0597 were combined (sensitivity = 84.49%, specificity = 94.57%), which was also considerably higher than the AUC of GAS5 (0.819; *P* < 0.001).

Finally, we evaluated whether plasma lnc-DC could be a potential novel biomarker for distinguishing LN (n = 77) from SLE without nephritis (n = 110). The AUC of lnc-DC was 0.670 (95% CI: 0.589-0.752; *P* < 0.001; sensitivity = 42.86%, specificity = 90.00%). To explore whether the combination of lnc-DC, GAS5 and linc0597 could improve the diagnostic efficiency, a binary logistic regression analysis was performed, the result showed that the AUC of combination of lnc-DC and GAS5 was 0.712 (95% CI: 0.638-0.787; *P* < 0.001), however, it has no significant difference compared with the AUC of lnc-DC (0.670; *P* =0.156).

### GAS5, linc0597 and lnc-DC expressions in patients with SLE, RA and SS

**Figure 3:**
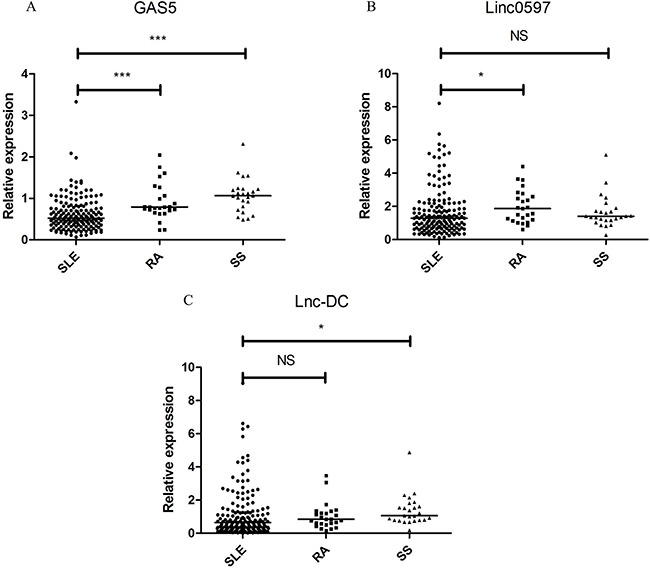
Relative expression of lncRNAs in the validation set of patients with SLE and disease controls Each symbol represents an individual subjects; horizontal lines indicate median values. **(A)** GAS5, **(B)** linc0597, **(C)** lnc-DC. SLE: systemic lupus erythematosus; RA: rheumatoid arthritis; SS: Sjögren's syndrome; NS: not significant; ^**^* *P*< 0.001, * *P*< 0.05.

The expression of GAS5 was decreased dramatically in patients with SLE patients compared with RA patients and SS patients (both *P* < 0.001); linc0597 levels was significantly lower in SLE patients than in RA patients (*P* = 0.039), but no significant difference was observed between SLE patients and SS patients (*P* = 0.505); the lnc-DC levels was significantly lower in SLE patients than in SS patients (*P* = 0.014), but no significant difference was found between SLE patients and RA patients (*P* = 0.428) (Figure 3).

**Figure 4:**
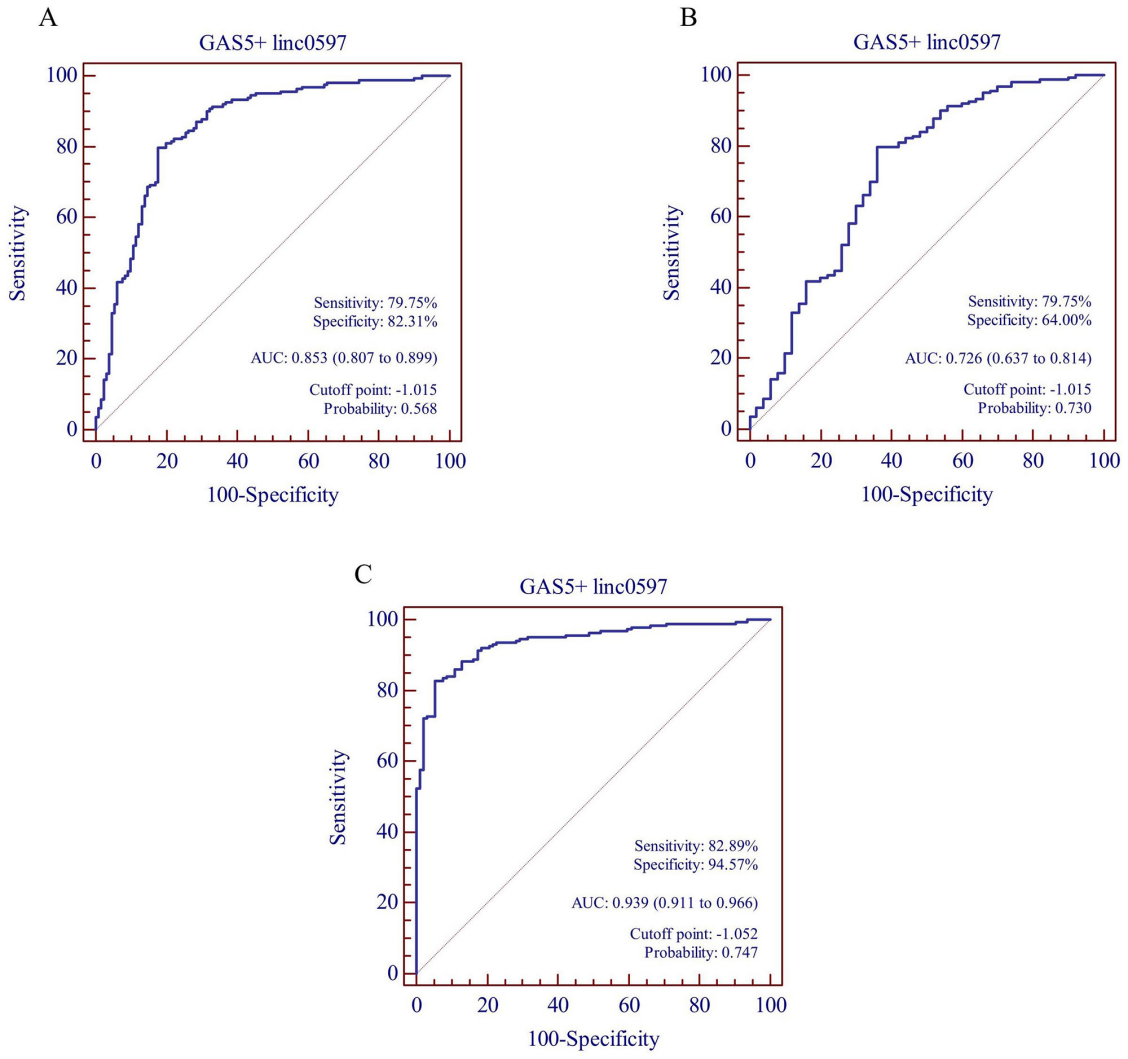
Receiver operating characteristic (ROC) curve analysis of GAS5 combined with linc0597 for the risk-score in **(A)** SLE patients in the validation set *vs* all controls (healthy controls in the validation set, RA and SS), **(B)** SLE patients in the validation set *vs* RA and SS, **(C)** combination set.

Subsequently, a risk score based on GAS5 and linc0597 from validation set was further assessed in SLE patients and all controls, the AUC for the risk score was 0.853 (95% CI: 0.807-0.899; *P* < 0.001; sensitivity = 79.75%, specificity = 82.31%). The risk score also significantly discriminated the patients with SLE from disease controls (RA and SS), and the AUC was 0.726 (95% CI: 0.637-0.814; *P* < 0.001; sensitivity = 79.75%, specificity = 64.00%). When the risk score was tested in all SLE patients and all healthy controls, the AUC for the risk score was 0.939 (95% CI: 0.911-0.966; *P* < 0.001; sensitivity = 82.89%, specificity = 94.57%) and no significant difference with 0.948 (Figure 4).

## DISCUSSION

In the current study, the results demonstrate higher expression of linc0597 and lower expression of GAS5 and lnc-DC in SLE patients. The observed down-regulation of plasma GAS5 was consistent with its expression in CD4 T-cells and B-cells from patients with SLE [31]. Furthermore, GAS5 was reported to induce apoptosis and growth arrest in human peripheral blood T-cells and increase apoptosis, which is central to the pathogenesis of SLE [32–34]. Conversely, Wu *et al*. showed that the SLE patients had lower expression of linc0949 and linc0597 in PBMCs when compared with RA patients or healthy controls. The increased linc0597 expression in plasma in SLE patients may thus be caused by increased cellular release of this lncRNA through increased exocytosis (leading to decreased intracellular content) and/or normal exocytosis of cells containing increased lncRNA. Li *et al*. indicated that linc0597 and linc0949 not only involved in innate immunity but also regulate the induction of proinflammatory cytokines like tumor necrosis factor (TNF)-α and interleukin (IL)-6 [35], both of which are implicated in SLE pathogenesis [36–39]. However, linc0949, which is present at low levels in PBMCs of patients with SLE, shows no significant difference between total SLE patients and healthy controls in our study. One explanation is that the lncRNA sequence length is very long, it is unlikely that lncRNAs exist in a full-length form in body fluids. The plasma lncRNAs probably exist in fragment form [29, 40]. Another possible explanation is that the expression of lncRNAs possess tissue specificity [41].

At present, there was no study investigating the plasma level of lnc-DC in any human disease. Our study showed that the level of plasma lnc-DC was significantly lower in SLE patients compared with healthy controls, and significantly higher in patients with LN when compared to SLE without nephritis. Lnc-DC, which is expressed exclusively in DCs, supports capacity of DCs to stimulate T cell activation. Wang *et al*. confirmed lnc-DC functions through activation of signal transducer and activator of transcription 3 (STAT3), an important transcription factor that regulates many immune associated genes. It has been proved that the STAT3 plays a crucial role in Th17 differentiation, T follicular helper and B cells, and STAT3 inhibition could represent a promising therapeutic target in SLE [42, 43]. Our results also show that the level of plasma GAS5 is correlated with disease activity and ESR, the level of plasma linc0597, lnc-DC and linc0949 are associated with the level of C3, and the level of plasma linc0949 correlated with disease activity and anti-dsDNA.

Recently, several studies have demonstrated the feasibility of using circulating cell-free miRNAs as potential biomarkers of SLE [10, 13, 44]. Similar to miRNA, emerging studies have shown great potential of circulating cell-free lncRNAs as powerful and non-invasive biomarkers in a number of diseases, including cancer, cardiovascular diseases [25, 26], renal diseases [45], gynecological disease [46], *etc*. Compared with miRNAs, lncRNAs show greater tissue specificity and complexity of biological functions, such as epigenetic regulation, enhancer-like function and alternative splicing, editing and export, and serve as small RNA precursors. Thus, lncRNAs as novel biomarkers have properties that are advantageous relative to miRNAs [25, 47]. Recently, Wu *et al*. showed that linc0949 in PBMCs could be a promising biomarker for diagnosis, disease activity evaluation and therapeutic response in SLE. However, the diagnostic performance of circulating lncRNAs in SLE has never been explored. Our study revealed that plasma GAS5 and linc0597 may serve as SLE-specific signature lncRNAs and could be used as candidate biomarkers of SLE. Combination of GAS5 and linc0597 from the logistic regression model demonstrated higher AUC (0.948) than respectively. The risk score based on GAS5 and linc0597 from validation set also demonstrated that it can also significantly discriminated the patients with SLE from RA and SS. When the patients with SLE were divided into LN and SLE without nephritis, the combination of lnc-DC and GAS5 with AUC 0.712 may server as potential novel biomarkers for distinguishing LN from SLE without nephritis.

However, several limitations in this study should be acknowledged. First, potential confounding factors should be considered in data interpretation, such as different clinical characteristics between screening and validation stage, different treatment strategies among patients, *etc*. Second, this study is limited by the patients from only two tertiary Hospitals, which may restrict the generalizability of our results. Third, this is a cross-sectional study, which also makes determining a causal relationship between lncRNAs and SLE challenging, thus further studies on the exact role of lncRNAs in SLE pathogenesis are needed. Fourth, due to the lack of data on ESR and C3 in health controls, we were unable to evaluate the values of lncRNAs plus ESR/C3 in SLE diagnosis.

In conclusion, our results provide novel empirical evidence that GAS5, linc0597 and lnc-DC may specifically identify patients with SLE, the combination of GAS5 and linc0597 could provide better diagnostic accuracy; in addition, lnc-DC may discriminate patients with LN from SLE without nephritis.

## MATERIALs AND METHODS

### Patients and healthy controls

A total of 187 patients with SLE were recruited between April 2015 and April 2016. All the patients with SLE were diagnosed according to the 1997 revised American College of Rheumatology (ACR) diagnostic criteria [48], which was used as the gold standard for diagnostic test of plasma lncRNAs in the current study. Renal involvement of SLE was defined according to the ACR criteria, i e, any one of the following: (i) persistent proteinuria ≥0.5 g/day; (ii) the presence of active cellular casts; or (iii) biopsy evidence of lupus nephritis. The disease severity was quantified according to the Systemic Lupus Erythematosus Disease Activity Index 2000 (SLEDAI-2K) [49]. Disease activity was quantified using the SLEDAI-2K score. More active SLE was defined as a SLEDAI-2K score >10, those patients with SLEDAI-2K ≤10 were classed as relatively inactive [15, 50]. The patients with rheumatoid arthritis (RA) were diagnosed according to the ACR/European League Against Rheumatism 2010 classification criteria [51]. The patients with Sjögren's syndrome (SS) were diagnosed in accordance with the revised 2002 American-European criteria [52]. Exclusion criteria of all patients were as follows: (i) patients with malignant tumours; (ii) patients with serious acute infection within six weeks before admission; (iii) patients complicated with other autoimmune disease; and (iv) patients suspected of drug or alcohol abuse.

The study subjects of first stage screening for selected lncRNAs were composed of 24 new-onset SLE patients (12 SLE without nephritis and 12 LN) and 12 age- and sex-matched healthy controls. New-onset SLE patients were identified when the following criteria were met: 1) first time diagnosis of SLE; 2) no history of corticosteroids or immunosuppressive drugs use before registration [53]. To validate the first stage screening results, we conducted a second stage evaluation of the candidate lncRNAs in an independent cohort consisting of 163 SLE patients (98 SLE without nephritis and 65 LN) and 80 age- and sex-matched healthy controls. The healthy controls were recruited from the physical examination center of the Second Affiliated Hospital of Anhui Medical University. To identify the specificity of candidate lncRNAs which were considered as potential biomarkers for SLE, we also detected their levels in 25 patients with RA and 25 patients with SS.

This study was approved by the Ethics Committee of Anhui Medical University. All participating subjects gave written consent according to the Declaration of Helsinki.

### Extraction of plasma

Peripheral blood (~5 mL) from all study subjects was collected into Ethylenediaminetetraacetic acid (EDTA)-anticoagulated tubes. The blood samples were centrifuged (1,500g for 10 minutes at 4°C, then 12000g for 10 minutes at 4°C) to obtain plasma. Plasma was then carefully removed, divided into aliquots and stored at -80°C until test.

### Total RNA isolation

Total RNA in 400ul plasma was isolated by using miRNeasy Mini Kit (Qiagen, Germany). The concentrations of RNA were measured using a NanoDrop™ 2000 spectrophotometer (Thermo Scientific, USA), approximately 200-600 ng of RNA was obtained from 400ul of plasma.

### Quantitative reverse transcription polymerase chain reaction (qRT-PCR)

Total RNA were reverse-transcribed into cDNA using a PrimeScript™ RT reagent kit (Takara Bio Inc, Japan). The quantitative real-time PCR (qPCR) was then carried out in duplicate on an ABI ViiA™ 7 Real-Time PCR System (Applied Biosystems, Foster City, CA, USA), using SYBR® Premix Ex Taq™ II (Takara Bio Inc, Japan) in 10 ul reactions containing 5 ul SYBR Green, 0.2 ul ROX Reference Dye II, 0.2-0.3 uM forward primer, 0.2-0.3 uM reverse primer, 2.2-2.4 ul sterile deionized warter and 2 ul cDNA. The relative expression level of lncRNA in plasma was normalized to the GAPDH expression [29, 54, 55]. All reactions were carried out in a 96-well optical plate at 95°C for 1 min, followed by 42 cycles at 95°C for 10 sec, 60°C for 30 sec and 72°C for 1 min, then melt curve were detected to confirm the specificity of amplification and lack of primer dimers. The primers used in qPCR of the lncRNAs are listed in Supplementary Table 1. After the reactions, the Ct values were determined using the fixed threshold settings.

The relative expression of lncRNAs were calculated using 2 -^ΔΔCt^ method normalized to endogenous control, with ^Δ^Ct = Ct_target_ − Ct_reference_, −^ΔΔ^Ct = − (sample ^Δ^Ct – control ^Δ^Ct) [56].

### Statistical analysis

The Kolomogorov-Smirnov test was used for checking normal distribution. Normally distributed data were expressed as means ± standard deviation (SD), whereas variables with a skewed distribution were presented as median (interquartile range) and log transformed to approximate normality before analysis. Categorical variables were represented by frequency and percentage. Student's *t*-test and chi-square test were used to determine whether the means and proportions of two groups are statistically different or not. One-way ANOVA were used for continuous variables with normal distributions for comparisons across multiple groups. The correlations between lncRNAs and clinical characteristics were analyzed using Spearman's rank correlation coefficient test. Logistic regression was used to calculate probabilities, odds ratios (OR) and corresponding 95% CI. Receiver operating characteristic (ROC) curves were constructed and the area under curve (AUC) was used to assess specificity and sensitivity of predictive power or feasibility of using plasma lncRNAs as biomarkers for SLE or LN. Positive predictive value (PPV) and negative predictive value (NPV) were calculated based on the Bayes theorem as follows:

$$\begin{array}{c} \text{PPV}=\frac{P\times Se}{P\times Se+(1-P)\times (1-Sp)}\\ \text{NPV=}\frac{(1-P)\times Sp}{\left(1-P)\times Sp+P\times (1-Se\right)} \end{array}$$

*P*: prevalence; *Se*: sensitivity; *Sp*: specificity

The prevalence of SLE for the PPV and NPV calculation was 36.03 per 100,000 persons, which was reported in our previous epidemiological survey. Accuracy was calculated by dividing the number of true positive and true negative by the number of the total population studied.

We assigned each sample a risk score using the weight by the regression coefficient that was estimated by the univariate logistic regression model from validation set. The risk score was calculated as follows: risk-score = (0.763×expression level of linc0597) + (−2.733×expression level of GAS5). *P*-values (two-tailed) ≤0.05 were considered statistically significant.

All statistical analyses were performed with the Statistical Package for the Social Sciences (SPSS) statistical software for Windows, Version 10.01 (SPSS Inc, IL, USA). Scatter diagrams were generated by GraphPad Prism version 5.01 (GraphPad Software, Inc, CA, USA) and the ROC curve analysis were performed with MedCalc version 11.4.2.0 (Mariakerke, Belgium).

## SUPPLEMENTARY MATERIALS FIGURES AND TABLES


