# Supplementary material for: Identification of long non-coding RNAs GAS5, linc0597 and lnc-DC in plasma as novel biomarkers for systemic lupus erythematosus
Source: Oncotarget. 2017 Feb 21;8(14):23650–63. doi: 10.18632/oncotarget.15569 (PMC5410334; doi:10.18632/oncotarget.15569)
Supplement: Supplementary file 1 [file oncotarget-08-23650-s001.pdf]

Identification of long non-coding RNAs GAS5, linc0597 and lnc-DC in plasma as novel biomarkers for systemic lupus erythematosus

SUPPLEMENTARY TABLES

Supplementary Table 1: Primers sequences used for qRT-PCR

Gene	Primers	Amplicon (bp)
GAPDH	F: 5’ GGGAAACTGTGGCGTGAT 3’<br>R: 5’ GAGTGGGTGTCGCTGTTGA 3’	299
GAS5	F:5’ TATGGTGCTGGGTGCGGAT 3’<br>R:5’ CCAATGGCTTGAGTTAGGCTT 3’	121
Linc0597	F:5’ TTGGATTCATCCCGTTCACCTCCA 3’<br>R:5’ CAGCATGACGATCAAGCGAGATTC 3’	121
Linc0949	F:5’ GCGAAGAGACCACCAAACAG 3’<br>R:5’ AAAGAAGCAGGACTACCCACT 3’	160
Lnc-DC	F:5’ GATCGTCATCCCTTCCTGG 3’<br>R:5’ GAAACAACCCCTCTTCCCTG 3’	139
HOTAIRM1	F:5’ AGGTTCCCGGAAGTCTGGC 3’<br>R:5’ CACTCCAAATCGGCCTTTGC 3’	70

F: Forward; R: Reverse

Supplementary Table 2: Correlation analysis the expression of plasma GAS5, linc0597, lnc-DC and linc0949 with categorical clinical parameters of SLE patients.

See Supplementary File 1

Supplementary Table 3: Associations of the expression of plasma GAS5, linc0597, lnc-DC and linc0949 with quantitative clinical parameters of SLE patients

Parameters	N	GAS5		Linc0597		Lnc-DC		Linc0949	
		r_s	*P* value	r_s	*P* value	r_s	*P* value	r_s	*P* value
C3	180	-0.118	0.115	-0.263	<0.001	-0.173	0.020	-0.158	0.034
C4	159	0.032	0.686	-0.060	0.449	-0.059	0.463	0.076	0.339
ESR	174	-0.230	0.002	0.037	0.628	0.051	0.507	-0.050	0.511
CRP	154	-0.094	0.246	0.036	0.660	0.020	0.801	0.127	0.116
SLEDAI-2K	187	-0.143	0.051	-0.094	0.198	-0.017	0.820	-0.166	0.023
age	187	0.020	0.783	0.098	0.183	0.084	0.254	0.077	0.294
duration	187	0.128	0.080	0.008	0.917	0.050	0.501	0.075	0.308

SLEDAI-2K: Systemic Lupus Erythematosus Disease Activity Index 2000; ESR: Erythrocyte Sedimentation Rate; CRP: C Reactive Protein; C3: Complement 3; C4: Complement 4
